# An assessment of the predictors of the dynamics in arable production per capita index, arable production and permanent cropland and forest area based on structural equation models

**DOI:** 10.1186/2193-1801-3-597

**Published:** 2014-10-11

**Authors:** Epule Terence Epule, Christopher Robin Bryant, Cherine Akkari, Mamadou Adama Sarr, Changhui Peng

**Affiliations:** Département de Géographie, Université de Montréal, Pavillon 520, ch. de la Côte-Sainte-Catherine, Local 332-3, CP 6128, succursale Centre-ville, Montréal, Québec H3C 3J7 Canada; Institute of Environmental Sciences, University of Quebec at Montreal (UQAM), CP 8888, succursale Centre-Ville, Montréal, QC H3C 3P8 Canada

**Keywords:** Arable production, Forest area, Arable production and permanent cropland, Structural equation modelling, Predictors

## Abstract

This study sets out to verify the key predictors of the dynamics of the arable production per capita index, the arable production and permanent crop land and forest area at a national scale in Cameroon. To achieve this objective, data for twelve time series data variables spanning the period 1961–2000 were collected from Oxford University, the United Nations Development program, the World Bank, FAOSTAT and the World Resource Institute. The data were analysed using structural equation models (SEM) based on the two stage least square approach (2SLS). To optimize the results, variables that showed high correlations were dropped because they will not add any new information into the models. The results show that the arable production per capita index is impacted more by population while the influence of rainfall on the arable production per capita index is weak. Arable production and permanent cropland on its part has as the main predictor arable production per capita index. Forest area is seen to be more vulnerable to trade in forest products and logging than any other variable. The models presented in this study are quite reliable because the p and t values are consistent and overall, these results are consistent with previous studies.

**Electronic supplementary material:**

The online version of this article (doi:10.1186/2193-1801-3-597) contains supplementary material, which is available to authorized users.

## Introduction

Assessing the feedbacks and predictors of the arable production per capita index, forest area and arable production and permanent cropland at a national scale in Cameroon is of great pertinence for several reasons. Agriculture contributes about 50% to the GDP in most African countries (FAO and UNIDO 2008). Primarily, in Cameroon, the agricultural sector provides between 55–60% of employment (INS 2009). By 2050, global agricultural production ought to increase by 70–100% if the increasing world population is to be adequately fed (Dubois 2011). However, most developing countries will witness a decline in agricultural production from about 3% to 1.2% over the period 2006 to 2050 (Bruinsma 2009). Secondly, many of the attempts to increase agricultural production in most developing countries in general and in Cameroon in particular have been based on the expansion of farmland in efforts to increase yields. Rosegrant and Cline (2003) and Rosegrant and Svendsen (1993) have argued that the expansion of farmland or cultivated area is a common method of increasing agricultural production to meet the rising food demand in most developing countries associated with low levels of intensification of agriculture. In most parts of the developing world, such land expansion is often at the expense of large areas of forest. A study by Epule et al. (2011) empirically justifies the conclusion that attempts at increasing arable farming yields in Cameroon have led to arable and permanent cropland being the second most vital cause of deforestation in Cameroon. Zhao et al. (2006) made similar observations for parts of Asia.

It can therefore be seen that the three variables being verified in this study are intricately related. In spite of this, there are currently no studies on Cameroon that have attempted to verify the key predictors of these variables using structural equation models (SEM). However, the state of research in this area is as follows: Yengoh and Ardo (2014) verified crop yield gaps in Cameroon using biophysical suitability modelling on specific crops; García-Ponce et al. (2012) attributed variations in agricultural productivity in Senegal to government policies; Amujoyegbe et al. (2010) and Kombiok et al. (2013) attribute crop yield declines in Cameroon and Ghana to declining soil fertility; Epule et al. (2011, 2012) used an empirically grounded regression model to assert that population growth is at the centre of forest area decline in Cameroon. While the latter study was able to determine causality, it is weakened because the multiple linear regression approach used only establishes the link between a single dependent variable and several independent variables; in this case, the feedbacks between the variables which mimic real life situations are absent (Petraitis et al. 1996). It is for this reason that this study employs the use of SEM. SEM determines the key predictors among a group of variables and is capable of modelling the feedback between several endogenous and exogenous variables, thus mimicking real life situations or representing the real world more adequately (Petraitis et al. 1996). To the best of our knowledge, this study is the first to adopt this approach in determining the predictors of the endogenous variables under consideration in Cameroon. Thus, this study will verify the predictors of the arable production per capita index, arable production and permanent cropland and forest area at a national scale in Cameroon. The approach of verifying the predictors of several endogenous variables using SEM is justified by the fact that it enhances prediction by introducing multi-way interactions among the endogenous and exogenous variables.

*Arable production per capita index in international* $ is used in this study in reference to the amount of food produced in relation to the population (i.e. per head) (FAO 2006). *Arable production and permanent cropland in hectares* is used in reference to the amount of food produced in relation to ecumene land that is viable for such production; in other words, it refers to food production based on the amount of arable land under cultivation (FAO 2006). *Forest area in hectares* refers to the amount of land covered by forest at a given point in time. In this study, *forest area* in hectares is assumed to decline at a rate of about 220 Kha/year (FAO 2006, 2010). The latter is the FAO’s estimate for the decade 1990–2000.

**Figure 1:**
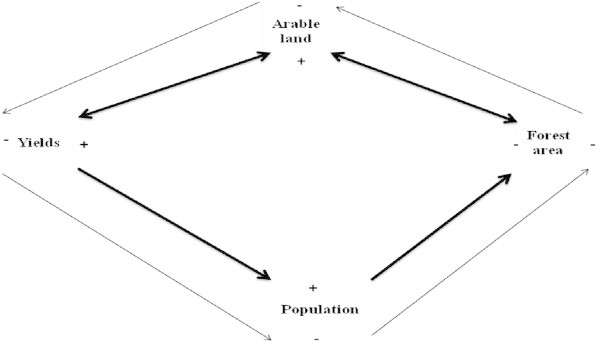
**Conceptualized schematic presentation of the interactions and feedbacks between the arable production per capita index, arable production and permanent cropland, forest area and population.**

Conceptually, it can be observed that when population growth increases the need for more food production becomes apparent. In most developing countries including Cameroon, arable farmers have relied for several centuries on farm land expansion in attempts to increase yields; unfortunately, this has been at the expense of large expanses of forest. The short term repercussions of such expansion are increased yields but in the long run, such yields are often not sufficient to meet population food needs (Figure 1). When arable land is increased, it provides a means for more production in the short term, but in the long term, expansion of arable land alone is not often sufficient to increase yields at proportions that should meet demand. When all the available land has been exploited, it becomes difficult to increase production further if agricultural intensification or agro-ecology methods cannot be used. On Figure 1 the inner thicker arrows represent the short term scenario with yield increase while the outer thinner arrows represent the declines in yields because of the inability to expand land further. The + and – signs show the nature of the effects on the variable.

## Study area and methods

### Study area

Cameroon is located in central Africa. More precisely, the country is located between latitude 2° N and 13° N of the equator. Longitudinally, Cameroon is located between longitude 8° E and 15° E of the prime meridian (Molua 2006). Cameroon has a total surface area of 475,400 km^2^ and a population of over 20.3 million people. Agriculture employs about 70–80% of the country’s population (Mundex Dataset 2012; Central Intelligence Agency 2012; Carr et al. 2005). Climatically, Cameroon has an equatorial climate in the south with rainfall levels of between 1500 mm-2000 mm per year and with an average annual temperature of about 25°C. In the north, the country has a tropical climate with annual rainfall dropping to as low as 400 mm around the Lake Chad basin region and equally high temperatures of about 28°C (Molua 2006).

### Data collection

To be able to explore the key predictors of the arable production per capita index, the arable and permanent cropland and forest area, twelve time series data variables (Table 1) spanning the period 1961–2000 were collected from various sources. The rainfall data were collected from the climate database of the School of Geography and Environment at Oxford University and the United Nations Development Program (http://www.geog.ox.ac.uk/research/climate/projects/undp-cp, United Nations Development Program 2014). The population data were collected from the World Bank, World Development Indicators database (http://www.google.com/publicdata, World Bank 2014). The remaining variables: arable production per capita index, arable production and permanent cropland, cattle stock, CO_2_ emissions, fuel wood, forest area, trade in forest products and logging, fertilizers, tractors-import value, tractors-quantity imported were retrieved from the Food and Agricultural Organization’s database (http://www.faostat.org, FAO 2014) and cross validated with those from the World Resources Institute’s data base (http://www.cait2.wri.org, World Resource Institute 2014).

**Table 1:** **Abbreviated and complete names of the twelve variables under study**

Abbreviated name	Complete variable name
ArablePCL	Arable production and permanent cropland (in ‘K’ Ha)
ArableProd	Arable production per capita index (international $)
CattleStock	Cattle stock (in ‘K’ heads)
CO2	Total CO_2_ emissions including land use change (in ‘K’ metric tons)
Fertilizer	Fertilizer consumption (in ‘K’ metric tons of nutrients)
ForestArea	Forest area (in ‘K’ Ha)
FuelWood	Fuel wood (in ‘K’ cubic metres)
POPS	Population
Rainfall	Rainfall (mm)
TractorImport	Agricultural tractors (quantity imported)
TractorVal	Agricultural tractors (import value in ‘K’ $)
Tradeforest	Trade in forest products/exports/logging (international $)

### Data analyses

Since the aim of this study was to verify the most important predictors of the arable production per capita index, arable production and permanent cropland and forest area, SEM based on the two stage least square (2SLS) approach was employed. This method can be rationalised by the fact that there are several endogenous variables and exogenous variables whose feedbacks in the system have to be determined in order to identify the actual predictors of the endogenous variables. The analyses were undertaken in the free R statistical software version 2.12.0. Three scenarios with structural simultaneous equations were identified. However, a test of the hypothesis that there are no significant correlations among the variables is required in order to assert that all the variables are suitable for the analysis. The entire procedure of performing the 2SLS method in R has been described by Henningsen and Hamann (2007). From the correlation analysis performed, variables that do not bring in any new information are those with high correlations and for the models to be optimized, such variables were removed from the analysis. In this case, CO_2_ emissions were removed because they have a correlation of 0.98 with population. Also, tractors-import values were removed because they have a correlation of 0.83 with tractors-quantity imported. In the paragraphs that follow, we describe the different scenarios, equations and the entire process of computing the predictors. The table that follows (Table 1) shows the abbreviated and complete names of all twelve variables. For a complete list of the time series data, see Additional file 1 section S1. The variables were abbreviated to facilitate handling in R. Furthermore, for the variables such as tractor (quantity imported) and tractors (import value) there were no data for the year 1978. This was dealt with by attributing to these variables the same measurements recorded in 1977 to avoid a gap and rationalized by the fact that these data points are rising along the series (i.e. 1800 and 19218 K$).

#### Scenario one

**Figure 2:**
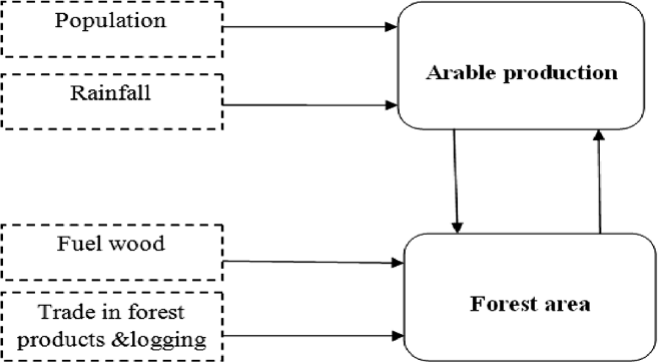
**Path diagram of the endogenous and exogenous variables used in Scenario one.**

Scenario one was structured to have as endogenous variables the arable production per capita index and forest area (Figure 2). As a result of this, two structural simultaneous equations are derived to determine the key predictors of the two endogenous variables. The equations are:

1

Where *Y*_*AP*_ is the arable production per capita index (endogenous variable); *α*_*1*_*X*_*FA*_ is forest area; *α*_*2*_*X*_*P*_ is population; *α*_*3*_*X*_*R*_ is rainfall (exogenous variables).

For the computation of these variables in the R inter face, the procedure below is used; however, for the detailed codes used in R, see Additional file 1 section S2:

The 2SLS estimated parameters are as follows:

Model Formula: ArableProd ~ ForestArea + Population + Rainfall +Instruments: ~Rainfall + Population + FuelWood + Tradeforest
2

Where *Y*_*FA*_ is forest area (endogenous variable); *β*_*1*_*X*_*AP*_ is arable production; *β*_*2*_*X*_*FW*_ is fuel wood; *β*_*3*_*X*_*TF*_ is trade in forest products and logging (exogenous variables).

For the computation of these variables in the R interface, the procedure below is used; however, for the detailed codes used in R, see Additional file 1 section S2:

The 2SLS estimated parameters are as follows:

Model Formula: ForestArea ~ ArableProd + FuelWood + Tradeforest +Instruments: ~Rainfall + population + FuelWood + Tradeforest

#### Scenario two

**Figure 3:**
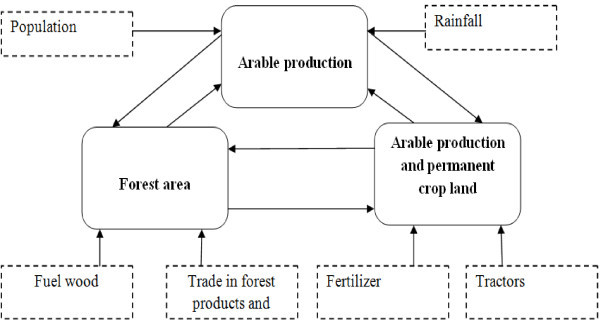
**Path diagram of the endogenous and exogenous variables used in Scenario two.**

Scenario two has been structured to have three key endogenous variables which are the arable production per capita index and forest area and arable production and permanent cropland (Figure 3). As a result of this, three structural simultaneous equations are derived to determine the key predictors of the three endogenous variables. The equations are:

3

Where: *Y*_*AP*_ is the arable production per capita index (endogenous variable); *α*_*1*_*X*_*FA*_ is forest area; *α*_*4*_*X*_*R*_*is* rainfall; *α*_*2*_*X*_*A_pcl*_ is arable and permanent cropland; *α*_*3*_*X*_*P*_ is population (exogenous variables).

For the computation of these variables in the R interface, the procedure below is used; however, for the detailed codes used in R, see Additional file 1 section S2:

The 2SLS estimated parameters are as follows:

Model Formula: ArableProd ~ ForestArea + ArablePCL + Population + Rainfall +Instruments: ~Rainfall + CattleStock +FuelWood + Tradeforest + Fertilizer + TractorImport
4

Where: *Y*_*FA*_*is* forest area (endogenous variable); *β*_*1*_*X*_*AP*_ is the arable production per capita; *β*_*2*_*X*_*A_pcl*_ is arable production and permanent cropland; *β*_*3*_*X*_*FW*_: is fuel wood; *β*_*4*_*X*_*TF*_ is trade in forest products and logging (exogenous variables).

For the computation of these variables in the R interface, the procedure below is used; however, for the detailed codes used in R, see Additional file 1 section S2:

The 2SLS estimated parameters are as follows:

Model Formula: ForestArea ~ ArableProd + ArablePCL + FuelWood + Tradeforest +Instruments: ~Rainfall + Population + FuelWood + Tradeforest + Fertilizer + TractorImport
5

Where: *Y*_*A*_*pcl*_ is arable production and permanent cropland (endogenous variable); *γ*_1_*X*_*FA*_ is forest area; *γ*_2_*X*_*AP*_ is the arable production per capita index; *γ*_3_*X*_*F*_ is fertilizers; *γ*_4_*X*_*T*_ is tractors (import value) (exogenous variables).

For the computation of these variables in the R inter face, the procedure below is used; however, for the detailed codes used in R, see Additional file 1 section S2:

The 2SLS estimated parameters are as follows:

Model Formula: ArablePCL ~ ArableProd + ForestArea + Fertilizer + TractorImport +Instruments: ~Rainfall + CattleStock + FuelWood + Tradeforest + Fertilizer + TractorImport

#### Scenario three

**Figure 4:**
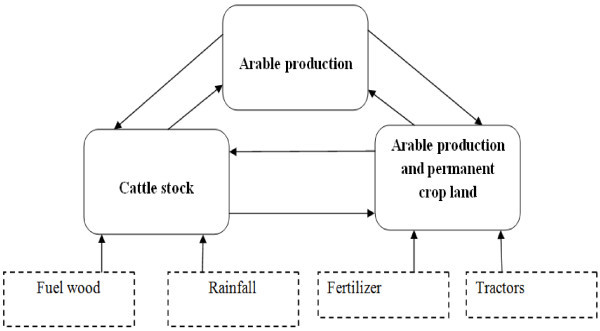
**Path diagram of the endogenous and exogenous variables used in Scenario three.**

Scenario three has been designed to have three key endogenous variables which are the arable production per capita index, cattle stock and arable production and permanent cropland (Figure 4). As a result of this, three structural simultaneous equations are derived to determine the key predictors of the three endogenous variables. The equations are:

6

Where: *Y*_*AP*_ is the arable production per capita index; *α*_1_*X*_*CS*_ is cattle stock; *α*_2_*X*_*A*_*pcl*_ is arable production and permanent cropland.

For the computation of these variables in the R inter face, the procedure below is used; however, for the detailed codes used in R, see Additional file 1 section S2:

The 2SLS estimated parameters are as follows:

Model Formula: ArableProd ~ ArablePCL + CattleStock + Instruments: ~Rainfall + FuelWood + Fertilizer + TractorImport
7

Where: *Y*_*CS*_ is cattle stock; *β*_1_*X*_*AP*_ is the arable production per capita index; *β*_3_*X*_*FW*_ is fuel wood; *β*_4_*X*_*RF*_ rainfall is rainfall; *β*_2_*X*_*A*_*pcl*_ is arable production and permanent cropland.

For the computation of these variables in the R interface, the procedure below is used, however, for the detailed codes used in R, see Additional file 1 section S2:

The 2SLS estimated parameters are as follows:

Model Formula: CattleStock ~ ArableProd + ArablePCL + FuelWood + Rainfall + Instruments: ~Rainfall + FuelWood +Fertilizer + TractorImport
8

Where: *Y*_*A*_*pcl*_ is the arable production and permanent cropland (endogenous variable); *γ*_2_*X*_*AP*_ is the arable production per capita; *γ*_1_*X*_*CS*_ is cattle stock; *γ*_3_*X*_*F*_ is fertilizers; *γ*_4_*X*_*T*_ is tractors.

For the computation of these variables in the R interface, the procedure below is used; however, for the detailed codes used in R, see Additional file 1 section S2:

2SLS Estimates

Model Formula: ArablePCL ~ ArableProd + CattleStock + Fertilizer + TractorImport +Instruments: ~Rainfall + FuelWood + Fertilizer + TractorImport

Once the above scenarios were set up and the data were analysed, a normal probability test was used to show whether process data exhibit the standard normal bell curve or the Gaussian distribution.

## Results and discussion

### Scenario one

For the results on the most important variable impacting the first endogenous variable in Scenario one (arable production: *Y*_*AP*_), represented by Equation (1), the SEM shows that population growth (*α*_*2*_*X*_*P*_) is the most significant determinant of (*Y*_*AP*_). This is supported by the fact that *α*_*2*_*X*_*P*_ has the highest t value and the lowest p value (<0.05) when compared to those of rainfall (*α*_*3*_*X*_*R*_) and forest area (*α*_*1*_*X*_*FA*_). In addition, the coefficient of *α*_*2*_*X*_*P*_ shows that a unit of change in *α*_*2*_*X*_*P*_ will produce 6.80 units of change in *Y*_*AP*_. Overall, we can say that when *α*_*2*_*X*_*P*_ increases, arable production reduces mainly due to the inelastic nature of arable production in the short run and the increase in pressure of population growth on land. *α*_*1*_*X*_*FA*_ is the second most significant variable and *α*_*3*_*X*_*R*_ is the least most significant variable (Table 2). By implication, it can be judged that the *α*_*1*_*X*_*FA*_ decline seen mainly through deforestation will have negative repercussions on Y_AP_ because of increased soil erosion and reduced soil organic carbon and organic nitrogen. The fact that the influence of *α*_*3*_*X*_*R*_ is least confirms the hypothesis that the influence of *α*_*3*_*X*_*R*_ in the food sovereignty scheme of Cameroon and most sub-Saharan African countries is weak in spite of rising rainfall in most of sub-Saharan Africa.

**Table 2:** **SEM outputs for Equation (**1**), Scenario one**

Exogenous variables	Coefficients	Standard Error	t-values	p-value	Rank of t-values
*α*_*1*_ *X*_*FA*_	-0.0065	0.00282	-2.32	0.02	2
*α*_*3*_ *X*_*R*_	0.15	0.13	1.10	0.27	3
*α*_*2*_ *X*_*P*_	-6.80	2.20	-3.06	0.004*	1

*α*_*1*_ *X*_*FA*_ forest area, *α*_*2*_ *X*_*P*_ population, *α*_*3*_ *X*_*R*_ rainfall; *most important predictor.

Considering the second endogenous variable in scenario one (*Y*_*FA*_), represented by Equation (2) of the SEM, it is observed that trade in forest products and logging (*β*_*3*_*X*_*TF*_) is the most significant determinant of forest area (*Y*_*FA*_). This is supported by the fact that *β*_*3*_*X*_*TF*_ has the highest t value and the lowest p value (<0.05) when compared to those of arable production (*β*_*1*_*X*_*AP*_) and fuel wood (*β*_*2*_*X*_*FW*_). The only anomaly is that instead of *β*_*3*_*X*_*TF*_, *β*_*1*_*X*_*AP*_ has the largest coefficient. This however does not imply *β*_*1*_*X*_*AP*_ is more significant because when the coefficients are divided by the standard errors, the t and p values remain the critical determinants of the most important variables because they consider both the coefficients and the standard deviation. In the latter situation, *β*_*3*_*X*_*TF*_ remains the most important determinant of *Y*_*FA*_. Overall, we can say that when *β*_*3*_*X*_*TF*_ increases, *Y*_*FA*_ decreases (Table 3).

**Table 3:** **SEM outputs for Equation** (2 ), **Scenario one**

Exogenous variables	Coefficients	Standard Error	t-value	p-value	Rank of t-value
*β*_*1*_ *X*_*AP*_	55.94	29.65	1.88	0.06	2
*β*_*2*_ *X*_*FW*_	-0.008	0.64	-0.01	0.98	3
*β*_*3*_ *X*_*TF*_	-0.01	0.0038	-3.55	0.0010*	1

*β*_*1*_ *X*_*AP*_ arable production, *β*_*2*_ *X*_*FW*_ fuel wood, *β*_*3*_ *X*_*TF*_ trade in forest products and logging;

*most important predictor.

### Scenario two

For the results regarding the most important variable impacting the first endogenous variable in Scenario two (arable production: *Y*_*AP*_), represented by Equation (3), the SEM shows that population growth (*α*_*3*_*X*_*P*_) is the most significant determinant of (*Y*_*AP*_). This is supported by the fact that *α*_*3*_*X*_*P*_ has the highest t value, the highest coefficient and the smallest p value when compared to those of rainfall (*α*_*4*_*X*_*R*_), forest area (*α*_*1*_*X*_*FA*_) and arable and permanent cropland (*α*_*2*_*X*_*A_pcl*_). In addition, the coefficient of *α*_*3*_*X*_*P*_ shows that a unit of change in *α*_*3*_*X*_*P*_ will produce 2.10 units of change in *Y*_*AP*_. Overall, we can say that when *α*_*3*_*X*_*P*_ increases, *Y*_*AP*_ reduces mainly due to the inelastic nature of *Y*_*AP*_ in the short run and the increased pressure of population growth on land. *α*_*1*_*X*_*FA*_ is the second most significant variable and *α*_*2*_*X*_*A_plc*_ is the third one while *α*_*4*_*X*_*R*_ is the least important (Table 4). The fact that the influence of *α*_*4*_*X*_*R*_ is least tends to confirm further the hypothesis that the influence of *α*_*4*_*X*_*R*_ in the food sovereignty scheme of Cameroon and most sub-Saharan African countries is weak.

**Table 4:** **SEM outputs for Equation** (3), **Scenario two**

Exogenous variables	Coefficients	Standard Error	t-value	p-value	Rank of t-value
*α*_*1*_ *X*_*FA*_	-0.03	0.0043	-7.59	0.05	2
*α*_*4*_ *X*_*R*_	-0.09	0.10	-0.95	0.82	4
*α*_*2*_ *X*_*A_pcl*_	-0.04	0.006	-6.75	0.34	3
*α*_*3*_ *X*_*P*_	-2.10	2.62	-8.16	0.02*	1

*α*_*1*_ *X*_*FA*_ forest area, *α*_*4*_ *X*_*R*_ rainfall, *α*_*2*_ *X*_*A_pcl*_ arable and permanent cropland, *α*_*3*_ *X*_*P*_ population;

*most important predictor.

Considering the second endogenous variable in Scenario two (*Y*_*FA*_), represented by Equation (4) of the SEM, it is observed that arable production and permanent cropland (*β*_*2*_*X*_*A_pcl*_) are the most significant determinants of forest area (*Y*_*FA*_). This is supported by the fact that *β*_*2*_*X*_*A_pcl*_ has the highest t value when compared to the other variables. The irregularities observed in the coefficients create anomalies and determines that the t values are the only reliable determinants of causality because the t value considers the coefficients and the standard deviation. Overall, we can say that when *β*_*2*_*X*_*A_pcl*_ increases, *Y*_*FA*_ decreases as the expansion of farm lands is often at the expense of forest area (Table 5).

**Table 5:** **SEM outputs for Equation** (4), **Scenario two**

Exogenous variables	Coefficients	Standard Error	t-value	p-value	Rank of t-value
*β*_*1*_ *X*_*AP*_	-21.85	4.26	-5.11	1.12	3
*β*_*2*_ *X*_*A _ pcl*_	-3.10	0.06	-45.16	0.15*	1
*β*_*3*_ *X*_*FW*_	-1.43	0.09	-15.85	1.49	2
*β*_*4*_ *X*_*TF*_	0.0008	0.0006	1.44	1.27	4

*β*_*1*_ *X*_*AP*_ arable production, *β*_*2*_ *X*_*A_pcl*_: arable production and permanent cropland,

*β*_*3*_ *X*_*FW*_ fuel wood, *β*_*4*_ *X*_*TF*_ trade in forest products and logging;

*most important predictor.

In the case of Equation (5) of scenario two, we observe that the most significant determinant of arable production and permanent cropland (*Y*_*A_pcl*_) is the arable production per capita index (*γ*_*2*_*X*_*AP*_). This is supported by the fact that *γ*_*2*_*X*_*AP*_ has the highest t value and the lowest p value. Generally, we expect that an increase in *γ*_*2*_*X*_*AP*_ will trigger a decline in *Y*_*A_pcl*_. In the same way, when *γ*_*1*_*X*_*FA*_ increases, *Y*_*A_pcl*_ will decrease (Table 6).

**Table 6:** **SEM outputs for Equation** (5), **Scenario two**

Exogenous variables	Coefficients	Standard Error	t-value	p-value	Rank of t-value
*γ*_1_ *X*_*FA*_	-0.18	0.01	-2.19	1.32	3
*γ*_2_ *X*_*AP*_	-6.63	3.03	-12.84	0.03*	1
*γ*_3_ *X*_*F*_	4.10	2.59	1.57	8.28	4
*γ*_4_ *X*_*T*_	0.02	0.003	6.58	0.12	2

*γ*_1_ *X*_*FA*_ forest area, *γ*_2_ *X*_*AP*_ arable production, *γ*_3_ *X*_*F*_ fertilizers, *γ*_4_ *X*_*T*_: tractors;

*most important predictor.

### Scenario three

As concerns Equation (6) in Scenario three, we observe that the most important determinant of arable production (*Y*_*AP*_) is cattle stock (*α*_1_*X*_*CS*_). This is seen as in this SEM equation, the latter has the highest t value and the lowest p value which is equally also less than 0.05. The overall implication of this equation is that *α*_1_*X*_*CS*_ reduces *Y*_*AP*_ because an increase in cattle rearing requires more land and often leads to a reduction in arable farmland since cattle rearing is often land dependent (Table 7). This is this case in the northern regions of Cameroon and parts of the North West and Western Highlands which constitute the cattle rearing hub of the country.

**Table 7:** **SEM outputs for Equation** (6), **Scenario three**

Exogenous variables	Coefficients	Standard Error	t-value	p-value	Ranks of t- value
*α*_1_ *X*_*CS*_	-0.006	0.002	-3.04	0.004*	1
*α*_2_ *X*_*A*_*pcl*_	0.005	0.004	1.09	0.279	2

*α*_1_ *X*_*CS*_ cattle stock, *α*_2_ *X*_*A*_*pcl*_ arable production and permanent crop land,

*most important predictor.

In the case of Equation (7), Scenario three, we observe that arable production and permanent cropland (*β*_2_*X*_*A*_*pcl*_) is the most influential variable affecting cattle stock (*Y*_*CS*_). It can be observed therefore that an increase in *β*_2_*X*_*A*_*pcl*_ will trigger an increase in *Y*_*CS*_ since more land will be established for crop production and animal rearing. Fuel wood is (*β*_3_*X*_*FW*_) seen as the second most important variable here and its influence is seen as it enhances *Y*_*CS*_ because when trees are cut to produce fire wood, more land becomes available for cattle rearing (Table 8).

**Table 8:** **SEM outputs for Equation** (7), **Scenario three**

Exogenous variables	Coefficients	Standard Error	t-value	p-value	Rank of t-value
*β*_1_ *X*_*AP*_	-9.00	6.34	-1.41	0.99	3
*β*_3_ *X*_*FW*_	0.49	0.06	7.67	0.16	2
*β*_4_ *X*_*RF*_	-0.00069	6.04	-0.0001	5.23	4
*β*_2_ *X*_*A*_*pcl*_	1.42	0.10	13.74	0.04*	1

*β*_1_ *X*_*AP*_ arable production, *β*_3_ *X*_*FW*_ fuel wood, *β*_4_ *X*_*RF*_ rainfall, *β*_2_ *X*_*A*_*pcl*_ arable production and permanent cropland; *most important predictor.

In the case of Equation (8), Scenario three, tractors (*γ*_4_*X*_*T*._) are seen as the most important variable affecting arable production and permanent cropland *Y*_*A*_*pcl*_.. This is evident from the very low p value and the high t value. It can be suggested that the more tractors the greater the *Y*_*A*_*pcl*_.. Also, arable production per capita index (*γ*_2_*X*_*AP*_) is the second most important variable (Table 9).

**Table 9:** **SEM outputs for Equation** (8), **Scenario three**

Exogenous variables	Coefficients	Standard Error	t-value	p-value	Rank of t-value
*γ*_2_ *X*_*AP*_	-59.27	1.30	-0.45	0.65	2
*γ*_1_ *X*_*CS*_	0.04	8.57	0.04	0.96	4
*γ*_3_ *X*_*F*_	12.52	3.03	0.41	0.68	3
*γ*_4_ *X*_*T*_	0.16	1.12	1.46	0.15*	1

*γ*_2_ *X*_*AP*_ arable production, *γ*_1_ *X*_*CS*_ cattle stock, *γ*_3_ *X*_*F*_: fertilizers *γ*_4_ *X*_*T*_ tractors;

*most important predictor.

**Figure 5:**
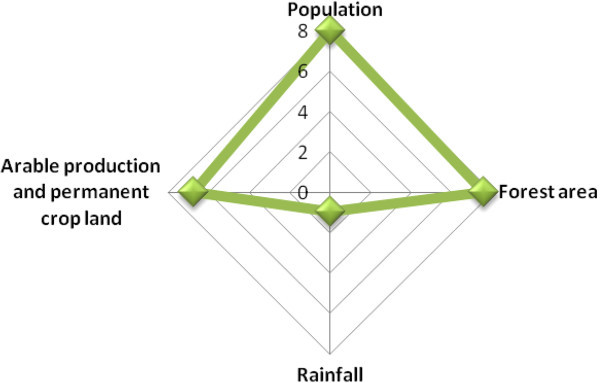
**Ranking of the four most significant variables affecting arable production in Cameroon based on the magnitude of t-values.**

It can be summarized from these scenarios that population is the most important predictor of the arable production per capita index. The second and third predictors in order of importance are forest area and arable and permanent crop land. Rainfall, however is seen as the weakest of all the variables under study (Figure 5). These results are consistent with several previous findings. For example, Alexandratos (2005, 2008) argues that rapid population growth impacts environmental resources because of the pressure of population on finite resources. This is similar to the reducing effect that population growth in Cameroon has on arable production. Borlaug (1999) also affirms the influence of a galloping world population as a major constraint on world food yields. There is also existing literature that is consistent with the influence of rainfall on food production in Cameroon and most of sub Saharan Africa is becoming weaker. For example, Olsson and Mryka (2008), Eklundh and Olsson (2003) and Hulme (2001) argue that the current trends in decline in food production in most of the Sahel and sub Saharan Africa cannot be attributed to rainfall which tends to be increasing but to the dynamics of various human land use processes. This constitutes a response to the question: If rainfall is increasing in most of the Sahel since the 1990s why does the region still face acute problems of food security?

**Figure 6:**
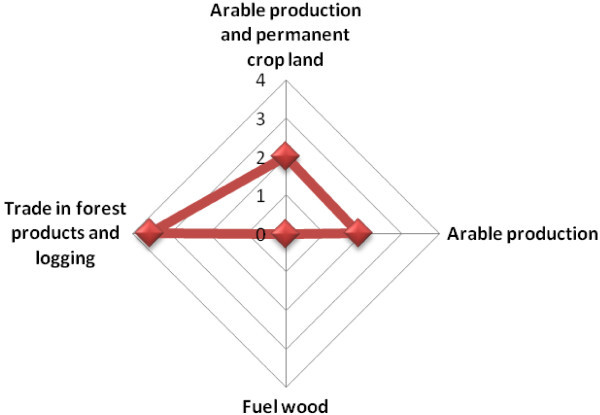
**Ranking of the four most significant variables affecting forest area in Cameroon based on the magnitude of t-values.**

With regards to the most important predictors of forest area dynamics, trade in forest products and logging concessions are the most important ones. Arable production and permanent cropland are second and third respectively (Figure 6). It has been argued that the increased profits obtained by timber companies and extractors of other forest products are key constraints on forest area decline in many parts of the world (Mertens and Lambin 1997; Carr et al. 2005; Vanclay 1993; Houghton 1991; Zhao et al. 2006). However, Angelsen and Kaimowitz (1999) argue that even when trade in forest products and logging are said to be dominant causes of forest area decline, population pressure is always at the centre of increased trade in forest products and logging. In the case of arable production and permanent cropland, it would be useful to consider how cropland expansion and cattle ranching have been able to produce forest area decline. In as much as this is true, the connection in all these studies is that as population grows, the need to feed more mouths increases and this often means more forest clearance.

Other factors that are considered important are the arable and permanent cropland as well as the arable production per capita index which are the second and third most valuable factors that explain deforestation in Cameroon. This result is highly consistent with other studies elsewhere. A study carried out in central Argentina reports that agricultural expansion for the purpose of cultivating Soya beans in particular is the main proximate cause of forest loss (Zak et al. 2008); this a view supported by Geist and Lambin (2002), when they report that about 96% of deforestation in Africa, Asia and Latin America is caused by agricultural expansion. As such, agricultural expansion remains a significant cause of deforestation and at times, it is related to population pressure. In support of this, it has been stated that one of the principal causes of deforestation in Panama has been the expansion of agricultural frontiers through extensive shifting cultivation systems.

**Figure 7:**
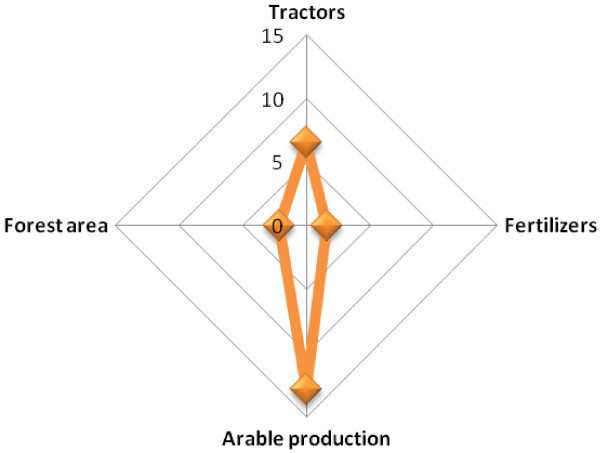
**Ranking of the four most significant variables affecting arable production and permanent cropland in Cameroon based on the magnitude of t-values.**

Finally, the most important predictors of arable production and permanent cropland in order of importance are the arable production per capita index, tractors, forest area and fertilizers (Figure 7). Agricultural expansion is also argued to be a key driver of arable and permanent cropland. This is seen as the incentives to cut more trees and establish more farmland are driven by arable production per capita. This is consistent with various studies (Zak et al. 2008; Geist and Lambin 2002; Angelsen and Kaimowitz 1999).

**Figure 8:**
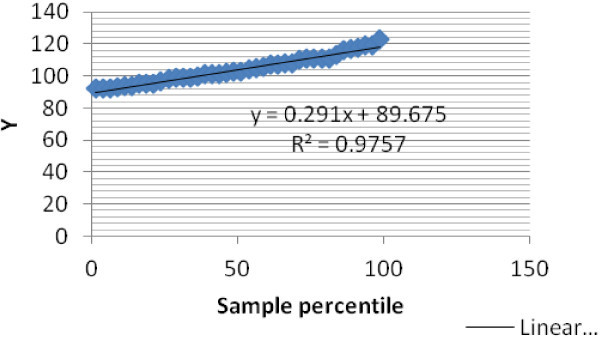
**Normal probability or quartile test results of the process data.**

To further verify the reliability of the data used in this study, a normal probability or quartile test was performed. This test is used to show whether process data exhibit the standard normal bell curve or Gaussian distribution or to tell how well the plotted points fit the normal line (Figure 8). If they fit well then, it can be assumed that the processed data are normally distributed. In our case, all the points fit the curve line adequately and an R^2^ of about 97% is obtained indicating a very high level of reliability.

## Conclusions

This study has demonstrated that the arable production per capita index is impacted more by population while the influence of rainfall on arable production is weak. The fact that the influence of rainfall is weak goes to show that the declining trends in production are more associated with human related land use activities. Furthermore, arable production and permanent cropland has as its main predictor arable production per capita. Forest area is seen to be vulnerable to trade in forest products and logging rather than to any other variable. This implies that, the loss in Cameroon’s forests are more linked to the commercialization of the forest than on the use of the forest for basic subsistence such as fuel wood collection by the local populations.

SEM models have been described as models that capture the feedbacks between several variables while determining the predictors of specific variables. While the aspect of introducing several endogenous and exogenous variables mimics reality, it in fact creates complexity that other models do not. As such, it is somehow difficult to use SEM to determine the predictors of a single endogenous variable because SEMs are structured to create diversity and consider several endogenous variables and instruments. As a result of this, this study observes that multiple linear regression models could be used when the objective is to create less diversity and to identify the predictors of only one dependent variable. Furthermore, it could be of pertinence if the vulnerability of specific crops to variables such as rainfall, population, fertilizers and machinery at the centre of interest. This would provide crop specific vulnerability information needed to inform policy. A meta analysis of the relative contributions of organic and conventional fertilizers on crop yields in Cameroon and Africa could also be valid alternatives for further research.

## Electronic supplementary material

Additional file 1: **Synthesis of the time series data of the twelve variables under used in this study.** (DOC 300 KB)
