# Supplementary material for: An assessment of the predictors of the dynamics in arable production per capita index, arable production and permanent cropland and forest area based on structural equation models
Source: Springerplus. 2014 Oct 11;3:597. doi: 10.1186/2193-1801-3-597 (PMC4447747; doi:10.1186/2193-1801-3-597)
Supplement: Supplementary file 1 — Additional file 1: Synthesis of the time series data of the twelve variables under used in this study. (DOC 300 KB) [file 40064_2014_1563_MOESM1_ESM.doc]

**Additional file 1 section S1:** Raw data of time series variables

| **Years** | | **Arable Production per capita index**  **( $)** | | **Forests ( 000 hectares)** | **Rainfall (mm)** | **Cattle stock ( in 000 heads)** | **Fuel Wood**  **( 000 cubic metres)** | | **Arable production and permanent crop land (in 000 hectares)** | **Population** | **Trade in forest products/exports/logging (in international $)** | **CO_2_ emissions (000metric tons)** | **Tractors(quantity imported** | **Tractors Import value(000$)** | **Fertilizer consumption(000 metric tons)** |
| --- | --- | --- | --- | --- | --- | --- | --- | --- | --- | --- | --- | --- | --- | --- | --- |
| 1961 | 97 | | 30907 | | 139.92 | 1750 | 6750 | 5510 | | 5524197 | 6148 | 26424.4 | 69 | 770 | 4 |
| 1962 | 101 | | 30687 | | 147.38 | 1750 | 6741 | 5570 | | 5646209 | 6304 | 27503 | 80 | 880 | 4 |
| 1963 | 107 | | 30467 | | 135.89 | 1850 | 6732 | 5620 | | 5774035 | 7416 | 28314.3 | 108 | 1110 | 5 |
| 1964 | 111 | | 30247 | | 138.54 | 1600 | 6724 | 5680 | | 5907535 | 9602 | 28975.6 | 95 | 1200 | 6 |
| 1965 | 101 | | 30027 | | 137.78 | 1740 | 6715 | 5740 | | 6046694 | 9899 | 29458.4 | 176 | 1940 | 8 |
| 1966 | 103 | | 29807 | | 146.59 | 1882 | 6706 | 5800 | | 6191813 | 12121 | 29771.4 | 90 | 974 | 12 |
| 1967 | 111 | | 29587 | | 132.48 | 1850 | 6697 | 5870 | | 6343314 | 13732 | 30110.1 | 120 | 1463 | 14 |
| 1968 | 113 | | 29367 | | 133.55 | 1900 | 6689 | 5920 | | 6501453 | 18330 | 30343.4 | 150 | 1648 | 17 |
| 1969 | 110 | | 29147 | | 150.73 | 2100 | 6680 | 5970 | | 6666501 | 20150 | 30565.7 | 450 | 3953 | 15 |
| 1970 | 116 | | 28927 | | 134.91 | 2325 | 6672 | 5978 | | 6838792 | 21305 | 30780.5 | 520 | 4570 | 20 |
| 1971 | 117 | | 28707 | | 147.64 | 2500 | 6694 | 6028 | | 7018240 | 22369 | 37929.5 | 360 | 3192 | 15 |
| 1972 | 119 | | 28505 | | 136.13 | 2325 | 6733 | 6095 | | 7205237 | 24533 | 39083.9 | 357 | 3174 | 13 |
| 1973 | 118 | | 28285 | | 133.43 | 2325 | 6731 | 6160 | | 7401135 | 55225 | 39959 | 850 | 8131 | 16 |
| 1974 | 123 | | 28065 | | 139.73 | 2400 | 6666 | 6280 | | 7607665 | 63928 | 40653.2 | 900 | 9245 | 17 |
| 1975 | 119 | | 27845 | | 134.91 | 2600 | 6594 | 6395 | | 7825971 | 44288 | 41420.5 | 420 | 4559 | 13 |
| 1976 | 111 | | 27625 | | 136.77 | 2750 | 6787 | 6510 | | 8056997 | 73315 | 41674.5 | 1300 | 13332 | 14 |
| 1977 | 111 | | 27405 | | 127.99 | 2917 | 6738 | 6644 | | 8300237 | 56535 | 42382.9 | 1800 | 19218 | 34 |
| 1978 | 108 | | 27185 | | 148.59 | 3000 | 6596 | 6671 | | 8553609 | 108463 | 42662.6 | 1800 | 19218 | 36 |
| 1979 | 107 | | 26965 | | 132.7 | 3100 | 6631 | 6912 | | 8814133 | 144583 | 48859.5 | 2050 | 24000 | 33 |
| 1980 | 106 | | 26745 | | 135.41 | 3681 | 6586 | 6930 | | 9079803 | 167628 | 49981.7 | 2500 | 25632 | 32 |
| 1981 | 104 | | 26525 | | 128.37 | 3782 | 6480 | 6960 | | 9349359 | 93848 | 53687.8 | 2300 | 18964 | 35 |
| 1982 | 105 | | 26305 | | 137.39 | 3512 | 6612 | 7010 | | 9623779 | 76696 | 56865.6 | 2500 | 15000 | 33 |
| 1983 | 102 | | 26085 | | 108.21 | 3431 | 6664 | 7060 | | 9902962 | 58143 | 62706.8 | 2400 | 12000 | 42 |
| 1984 | 99 | | 25865 | | 125.48 | 3561 | 6730 | 7110 | | 10200049 | 60055 | 63152.3 | 2482 | 9623 | 48 |
| 1985 | 103 | | 25645 | | 132.58 | 4151 | 6763 | 7160 | | 10508768 | 97701 | 63411.1 | 3741 | 12274 | 49 |
| 1986 | 108 | | 25425 | | 122.49 | 4255 | 6778 | 7230 | | 10832848 | 60043 | 66258 | 2436 | 11407 | 45 |
| 1987 | 92 | | 25205 | | 117.26 | 4362 | 6987 | 7210 | | 11170539 | 73762 | 65413.5 | 1692 | 9287 | 50 |
| 1988 | 95 | | 24985 | | 130.95 | 4471 | 7265 | 7210 | | 11519006 | 112673 | 61710.8 | 1208 | 8206 | 38 |
| 1989 | 95 | | 24765 | | 123.45 | 4582 | 7388 | 7190 | | 11874217 | 99833 | 64686.4 | 1480 | 10421 | 28 |
| 1990 | 93 | | 24545 | | 129.5 | 4697 | 7648 | 7170 | | 12232973 | 265451 | 61490 | 1309 | 9517 | 22 |
| 1991 | 94 | | 24325 | | 130.41 | 4700 | 7887 | 7150 | | 12594697 | 234532 | 61429.3 | 150 | 1342 | 18 |
| 1992 | 92 | | 24105 | | 144.46 | 4700 | 8087 | 7160 | | 12959446 | 295604 | 73131.4 | 580 | 4000 | 21 |
| 1993 | 92 | | 23885 | | 136.7 | 4700 | 8382 | 7160 | | 13325466 | 209312 | 79107 | 305 | 2714 | 23 |
| 1994 | 95 | | 23665 | | 134.45 | 4700 | 8624 | 7160 | | 13690847 | 299061 | 81111 | 370 | 3273 | 30 |
| 1995 | 98 | | 23445 | | 130.41 | 4650 | 8722 | 7160 | | 14054403 | 434121 | 82007.4 | 310 | 2550 | 30 |
| 1996 | 101 | | 23225 | | 137.1 | 4623 | 8819 | 7160 | | 14415168 | 424069 | 82137.9 | 131 | 3430 | 34 |
| 1997 | 94 | | 23005 | | 137.6 | 4737 | 8915 | 7160 | | 14773789 | 436161 | 82936.3 | 85 | 1398 | 38 |
| 1998 | 99 | | 22785 | | 128.45 | 4846 | 8957 | 7160 | | 15132782 | 268232 | 83502.9 | 300 | 4498 | 39 |
| 1999 | 100 | | 22565 | | 137.37 | 5500 | 9033 | 7160 | | 15495758 | 401790 | 83696.5 | 173 | 1752 | 49 |
| 2000 | 99 | | 22345 | | 128.12 | 5882 | 9111 | 7160 | | 15865456 | 435640 | 84105.5 | 292 | 4567 | 47 |

**Additional file 1 section S2:** Codes used in building SEM

## Consider Time Series present in the data

## Store APC data as a time series

APCtime = ts(ArableProd)

APCtime

## Take a look at the data

par(mfrow=c(2,1))

# Plot the time series data

plot.ts(APCtime)

# plot the correlogram

acf(APCtime)

# Test the hypothesis that there are no significant correlations

# up to lag of 10

Box.test(APCtime, lag=10, type="Ljung-Box")

## Create a correlation matrix for the data

cor(raw_dat)

## Load the library required to build SEM models.

library(sem)

scenario1.eqn1 <- tsls (ArableProd~ForestArea+Pops+Rainfall)

+instruments=~Rainfall+CattleStock+FuelWood+Tradeforest, data=raw_dat)

> summary(scenario1.eqn1)

The 2SLS estimate parameters are as follows:

Model Formula: ArableProd ~ ForestArea + Population + Rainfall

+Instruments: ~Rainfall + Population + FuelWood + Tradeforest

Scenario1.eqn2 <- tsls(ForestArea~ArableProd+FuelWood+Tradeforest)

+instruments=~Rainfall+pops+FuelWood+Tradeforest, data=raw_dat)

> summary(scenario1.eqn2)

The 2SLS estimate parameters are as follows:

Model Formula: ForestArea ~ ArableProd + FuelWood + Tradeforest

+Instruments: ~Rainfall + population + FuelWood + Tradeforest

Scenario2.eqn1 <- tsls

(ArableProd~ForestArea+ArablePCL+pops+Rainfall)

+instruments=~Rainfall+Pops+FuelWood+Tradeforest+Fertilizer+TractorImport, data=raw_dat)

> summary(scenario2.eqn1)

The 2SLS estimate parameters are as follows:

Model Formula: ArableProd ~ ForestArea + ArablePCL + Population + Rainfall

+Instruments: ~Rainfall + CattleStock + FuelWood + Tradeforest + Fertilizer + TractorImport

Scenario2.eqn2 <- tsls(ForestArea ~ArableProd+ArablePCL+FuelWood+Tradeforest)

+instruments=~Rainfall+Population+FuelWood+Tradeforest+Fertilizer+TractorImport, data=raw_dat)

> summary(scenario2.eqn2)

The 2SLS estimate parameters are as follows:

Model Formula: ForestArea ~ ArableProd + ArablePCL + FuelWood + Tradeforest

+Instruments: ~Rainfall + Population + FuelWood + Tradeforest + Fertilizer + TractorImport

Scenario2.eqn3 <- tsls(ArablePCL ~ ArableProd+ForestArea+Fertilizer+TractorImport)

+ instruments=~Rainfall+CattleStock+FuelWood+Tradeforest+Fertilizer+TractorImport, data=raw_dat)

> summary(scenario2.eqn3)

The 2SLS estimate parameters are as follows:

Model Formula: ArablePCL ~ ArableProd + ForestArea + Fertilizer + TractorImport

+Instruments: ~Rainfall + CattleStock + FuelWood + Tradeforest + Fertilizer + TractorImport

Scenario3.eqn1 <- tsls(ArableProd ~ ArablePCL+CattleStock)

+ instruments=~Rainfall+FuelWood +Fertilizer+TractorImport, data=raw_dat)

> summary(Scenario3.eqn1)

The 2SLS estimate parameters are as follows:

Model Formula: ArableProd ~ ArablePCL + CattleStock

+Instruments: ~Rainfall + FuelWood + Fertilizer + TractorImport

> Scenario3.eqn2 <- tsls(CattleStock ~ ArableProd+ArablePCL+FuelWood+Rainfall)

+ instruments=~Rainfall+FuelWood +Fertilizer+TractorImport, data=raw_dat)

> summary(Scenario3.eqn2)

The 2SLS estimate parameters are as follows:

Model Formula: CattleStock ~ ArableProd + ArablePCL + FuelWood + Rainfall

+Instruments: ~Rainfall + FuelWood + Fertilizer + TractorImport

Scenario3.eqn3 <- tsls(ArablePCL ~ ArableProd+CattleStock+Fertilizer+TractorImport)

+ instruments=~Rainfall+FuelWood +Fertilizer+TractorImport, data=raw_dat)

> summary(Scenario3.eqn3)

2SLS Estimates

Model Formula: ArablePCL ~ ArableProd + CattleStock + Fertilizer + TractorImport

+Instruments: ~Rainfall + FuelWood + Fertilizer + TractorImport

**Additional file 1 section S3:** Figures not included in text

**
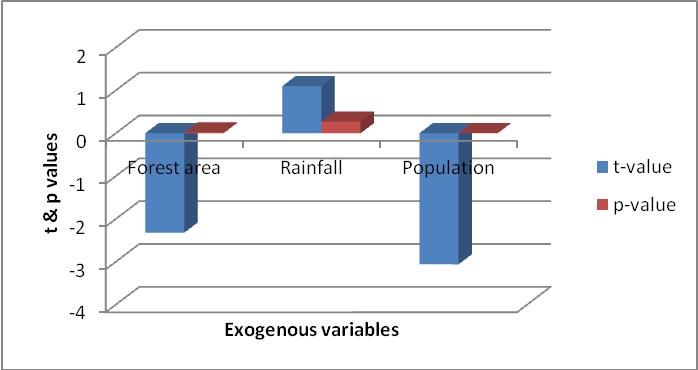
**

**Figure S1.** Exogenous variables, p-values and t-values used in equation one, Scenario one. Note that the significance of a variable is based on magnitude and the signs only provide an indication of the direction of the effect on the endogenous variable.

**
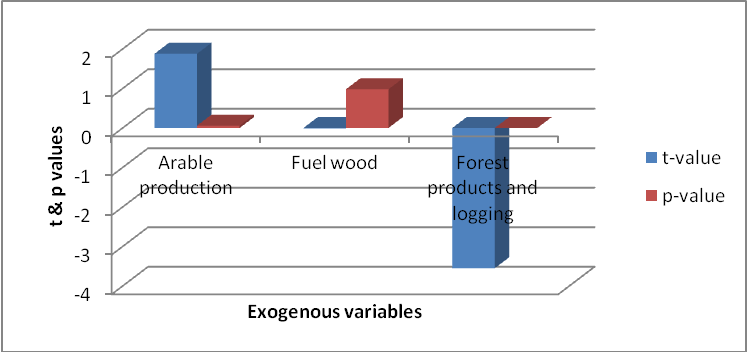
**

**Figure S2.** Exogenous variables, p-values and t-values used in equation two, Scenario one. Note that the significance of a variable is based on magnitude and the signs only provide an indication of the direction of the effect on the endogenous variable.

**
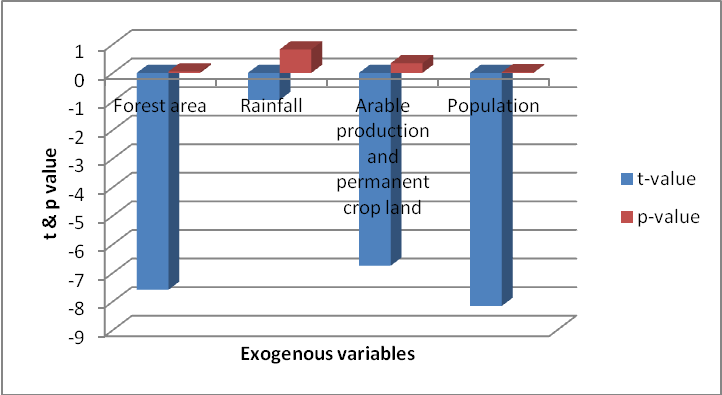
**

**Figure S3.** Exogenous variables, p-values and t-values used in equation one, Scenario two. Note that the significance of a variable is based on magnitude and the signs only provide an indication of the direction of the effect on the endogenous variable.


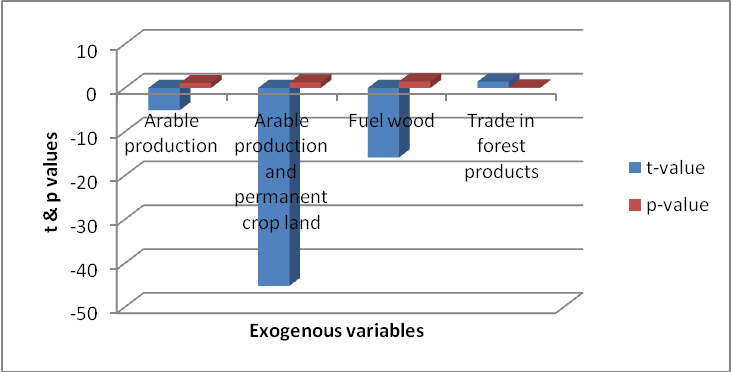


**Figure S4.** Exogenous variables, p-values and t-values used in equation two, Scenario two. Note that the significance of a variable is based on magnitude and the signs only provide an indication of the direction of the effect on the endogenous variable.

**
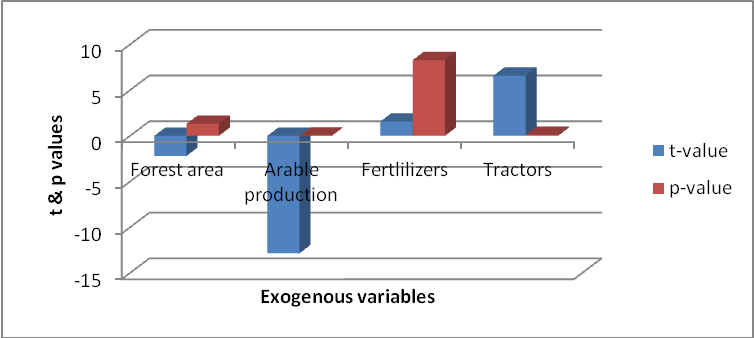
**

**Figure S5.** Exogenous variables, p-values and t-values used in equation three, Scenario two. Note that the significance of a variable is based on magnitude and the signs only provide an indication of the direction of the effect on the endogenous variable.

**
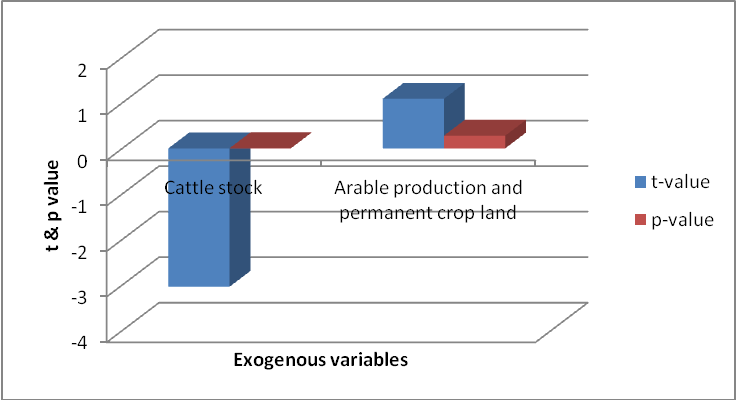
**

**Figure S6.** Exogenous variables, p-values and t-values used in equation one, Scenario three. Note that the significance of a variable is based on magnitude and the signs only provide an indication of the direction of the effect on the endogenous variable.

**
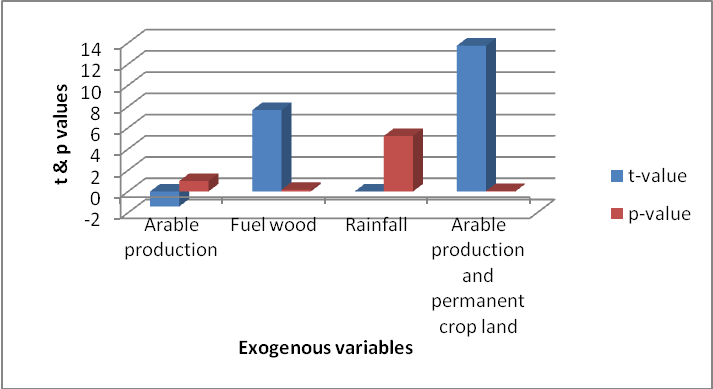
**

**Figure S7.** Exogenous variables, p-values and t-values used in equation two, Scenario three. Note that the significance of a variable is based on magnitude and the signs only provide an indication of the direction of the effect on the endogenous variable.

**
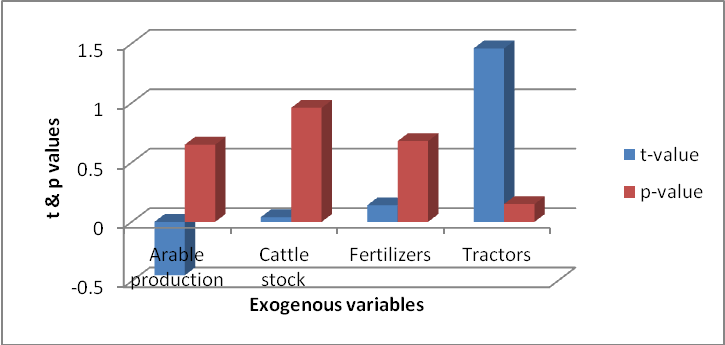
**

**Figure S8.** Exogenous variables, p-values and t-values used in equation three, Scenario three. Note that the significance of a variable is based on magnitude and the signs only provide an indication of the direction of the effect on the endogenous variable.
